# Can Functional Motor Capacity Influence Mortality in Advanced Chronic Kidney Disease Patients?

**DOI:** 10.3390/nu16162689

**Published:** 2024-08-13

**Authors:** Ángel Nogueira-Pérez, Paloma Ruiz-López-Alvarado, Guillermina Barril-Cuadrado

**Affiliations:** 1 Avericum, 35220 Las Palmas, Spain; 2 Department of Nephrology, Hospital Universitario de la Princesa, 28006 Madrid, Spain; paloma.ruiz.pr1@gmail.com; 3 Fundación Investigaciones Bioimédicas, 28290 Las Rozas de Madrid, Spain

**Keywords:** advanced chronic kidney disease, functional capacity, short physical performance battery, mortality

## Abstract

Alterations in the body’s nutritional status or composition may be observed as the kidney disease advances, which could influence the kidney’s functional capacity and, consequently, could increase the risk of mortality. The aim of the study is to determine the influence of functional capacity on mortality assessed by different functional tests in patients with advanced chronic kidney disease (ACKD). A prospective observational study was designed, which included 225 patients followed for 8 years in a CKD clinic. The study assessed functional capacity by using a range of tests, which included the Short Physical Performance Battery, the 6 minutes walking gait test, the timed up and go, and the four versions of the sit-to-stand test. Additionally, body composition and nutritional conditions were considered, taking into consideration various biochemical indicators such as albumin, prealbumin, c-reactive protein (CRP), lymphocytes, and transferrin, muscle strength, comorbidity, and frailty. The relationship between functionality and all-cause mortality was investigated using a Cox proportional hazard model. A total of fifty patients died during the duration of the study. Patients who performed worse on the function and muscle strength tests showed a worse body composition and nutritional status, and exhibited a reduced life expectancy. Inflammation (CRP) was associated with an increased risk of mortality (model 1: hazard ratio (HR) = 1.246; 95% confidence interval (95% CI = 1.014–1.531; model 2: HR = 1.333; 95% CI = 1.104–1.610). Good functional capacity as determined by the SPPB test decreased the risk of mortality (model 1: HR = 0.764; 95% CI = 0.683–0.855; model 2 HR = 0.778; 95% CI = 0.695–0.872). Cut-off points of maximum sensitivity and specificity for mortality were obtained with different tests. The study demonstrated that functional capacity influences mortality in patients with ACKD, being higher in those patients with impaired functionality regardless of the test used, although the SPPB allows a larger number of patients to be assessed. Therefore, it is essential to incorporate the assessment of functionality into the comprehensive care of patients with CKD.

## 1. Introduction

The percentage of people with chronic kidney disease (CKD) is currently on the rise, with an estimated worldwide prevalence of >10% [1,2]. The decrease in kidney function can be seen in individuals of all ages, although it is more prevalent in individuals who are 65 years old or older [3]. Consequently, chronic kidney disease (CKD) is now recognized as a condition mostly affecting the elderly [4].

As age progresses, changes in body composition are observed, such as a decrease in fat-free mass, especially muscle mass, and an increase in both subcutaneous and visceral fat mass [5,6]. These changes in body composition lead to an increased risk of frailty, which favors a decrease in functional capacity [6,7,8]. Functional capacity refers to the capability to carry out fundamental activities of daily life without experiencing pain or fatigue [9,10].

CKD is characterized by the existence of long-term structural or functional damage to the kidneys, persisting for a minimum of three months, and having a noticeable effect on the patient’s overall health. The disease is categorized into five stages according to the computed glomerular filtration rate, which is determined using creatinine as a metric. Stages 3B, 4, and 5 are classified as advanced stages, thus indicating ACKD.

This decrease in functional capacity may have its origin in sedentary lifestyles and poor eating habits in combination with muscle mass loss. These factors favor the appearance of sarcopenia and cachexia, which in turn increase the risk of mortality or the unscheduled start of renal replacement therapy in patients considered as candidates [10,11,12]. Ensuring a sufficient intake of protein and calories, in accordance with the nutritional guidelines for individuals with renal conditions, and tailoring exercise practices to the patient’s specific characteristics are crucial for maintaining optimal nutritional status and promoting overall functionality.

While there is presently no established protocol specifically designed for evaluating functional capacity in patients with CKD, it is possible to modify general functional capacity tests for use in these patients. Given the higher prevalence of CKD in elderly patients, it would be prudent to utilize validated techniques specifically designed for geriatric patients. Nevertheless, due to the diverse nature of the population affected by renal disease, it is necessary to integrate several techniques. Relying just on a single test may prove to be inadequate in encompassing the whole range of patients [12,13,14,15].

The aim of this study was to determine the influence of functional capacity on mortality in patients with ACKD.

## 2. Materials and Methods

Our work comprises a longitudinal retrospective observational analysis to evaluate the 8-year survival of 225 patients from a single multidisciplinary ACKD center.

The study encompassed all patients, aged 18 to 95, female and male, who had advanced stages 3b–5 of CKD and were not undergoing dialysis. Additionally, participants were required to be capable of doing the Short Physical Performance Battery (SPPB) test. Patients who were unable to accomplish the task were excluded. The study encompasses a group of patients from November 2011 to September 2022. In the mortality analysis, patients who initiated renal replacement therapy were considered as having been lost to follow-up. Patients participated in a study to assess their functional capacity and muscle strength using hand grip strength, body composition using bioimpedance, and nutritional status evaluation. All data have been extracted and recorded in the clinical history of the patients in the ACKD unit, where the protocol mandates an annual assessment of functional capacity.

### 2.1. Study of Functional Capacity and Muscle Strength

#### 2.1.1. Functional Capacity Was Assessed Using the Following Tests

##### Short Physical Performance Battery Test (SPPB)

A functional capacity test widely used in geriatrics that assesses functionality by means of 3 short tests [16,17]: (1) Balance test: This consists of assessing if the patient is able to maintain their balance for at least 10 s in a position with parallel feet, semi-tandem feet and tandem feet. (2) Four meter test: This consists of measuring the time the patient takes to walk four meters. The score will be graded according to different time ranges. (3) 5STS test: This consists of measuring the time it takes the patient to stand up and sit down from a chair 5 times without leaning on it.

##### 6-Minute Walk Test (6MWT)

This consists of measuring the distance a patient is able to walk in 6 min. In our study it was carried out in a 30-meter corridor. Once the test begins the patient is told to walk and to stop in the event of any setback. The examiner does not walk next to the patient so that they do not adjust their pace. Once the test is completed, the number of times the patient walked down the corridor is multiplied by the corridor length to obtain the total distance traveled [18,19].

##### Timed Up and Go Test (TUTG)

This consists of the patient getting up from a chair which measured approximately 46 cm in height, walking 3 m and sitting down again in the same chair. This was performed twice, once as a test and a second time in which the measurement was recorded with a stopwatch [20].

##### Sit-to-Stand Test (STS5)

This consists of the patient standing up and sitting down from a chair without leaning on it. There are different versions of this test, and in our study we used four versions: the STS5 and STS10, which consist of measuring the time that the patient takes to stand up and sit down from a chair 5 and 10 times, respectively, and the STS30 and STS60, which consist of counting the number of squats that the patient performs in 30 and 60 s [21,22,23].

#### 2.1.2. Muscle Strength Was Assessed with the Hand Grip Test

Hand grip strength (HGS): This is a pressure force measurement, which can be performed either sitting or standing. In our study it was performed standing up, in a neutral position with the arm at 90°. Three measurements were made, from which only the highest value was taken into consideration [24,25].

### 2.2. Comorbidity

Comorbidity was determined using the Charlson index, which consists of a list of 19 items or pathologies with a score determined according to severity. The patient’s age is taken into consideration, adding one point for each decade of age beyond the age of 50. The total obtained classified the patient as having none, low, or high comorbidity. This index also evaluates the 10-year mortality risk [26].

### 2.3. Frailty

Fried’s criteria was chosen to determine frailty [27]. It includes the following five items: (1) unintentional weight loss; (2) patient’s fatigue, assessed through 2 questions of the Centre for Epidemiological Studies Depression Scale (CES-D); (3) muscle weakness, assessed with hand grip strength measure adjusting the result for age and sex; (4) decrease in gait speed (5) decrease in physical activity. To diagnose frailty, at least 3 of the 5 criteria must be met.

### 2.4. Nutritional Status Study

#### 2.4.1. Body Composition Study: Bioimpedance

This was performed with a single-frequency electrical bioimpedance analyzer (BIA), measuring at 50 kHz and 800 μA. The model used was Body Impedance Analyzer BIA-101, Akern-RJL systems, Florence, Italy. Bioimpedance is a non-invasive, non-observer-dependent method to estimate body hydration, composition parameters, and nutritional status. It measures the opposing force exerted by the different cells and tissues of the organism when alternating electric current passes through them. This measurement is performed by placing the patient in a supine position with their arms 30° apart and their legs 45° apart. The circuit is made by placing two pairs of electrodes, one on the back of the hand and another one on the back of the foot, about 5 cm apart [28].

We also measured Phase angle (PA), Body Cell Mass (BCM), Total Body Water (TBW) Na/k interchangeable, Intracellular Body Watter (IBW), Extracellular Body Water (EBW), Body Cell Mass Index (BCMI), and Appendicular Skeletal Muscle Mass.

Non anthropometric measures were also considered for this study.

#### 2.4.2. Laboratory Parameters

For the nutritional study, biochemical data were collected from the patient’s clinical history such as albumin, prealbumin, CRP, lymphocytes, and transferrin levels. Furthermore, S-albumin was measured using the colorimetric standard method (Roche/Hitachi 904^®^/Modular ACN413, Roche Diagnostics, Basel, Switzerland) and the bromocresol green method and s-Prealbumin and s-CRP were measured using immunoturbidimetry methods (Roche/Hitachi 904^®^/Model P: ACN 218, Roche Diagnostics, Basel, Switzerland) [29].

### 2.5. Statistical Analysis

We conducted a Kolmogorov–Smirnov normality test as part of our statistical analysis. Continuous variables were quantified using the mean and standard deviation. The *t*-test for independent samples was employed to assess the differences between two groups, while Anova was utilized to compare means across three or more groups. Categorical variables were represented as integers and percentages and were evaluated using either the chi-square test or Fisher’s exact test.

The mortality study utilized Kaplan–Meier plots to assess the cumulative survival rate based on various functional tests. A Cox proportional hazard model was used to examine the association between functionality and all-cause mortality. We created two multivariable proportional hazard models. A multivariate Cox proportional hazards regression was conducted by selecting covariates and clinically prominent variables with *p <* 0.05 in univariate Cox analysis. Model 1 was adjusted for age, gender, Charlson comorbidity index, and biochemical parameters. Anthropometric parameters were incorporated into Model 2 to make adjustments.

The cut-off points for mortality of the different variants of the STS test and functional tests were determined by means of receiver operating characteristic (ROC) curve analysis, using the SPPB test as a reference method.

Statistical significance was set at *p* < 0.05.

Data were analyzed using the statistical software IBM Corp. Released 2015. IBM SPSS Statistics for Windows, Version 23.0. Armonk, NY, USA: IBM Corp.

## 3. Results

### 3.1. General Characteristics of the Study Population

A total of 225 ACKD patients were included in the study. Of them, 148 were male (65.8%). Mean age was higher in the female group, although the difference was not statistically significant. Patients were classified into age groups (15), (<55, 55–64, 65–74, 75–84, ≥85 years), with the 75–84 year age group being the biggest one. Patients presented a high comorbidity score determined by the Charlson index (>6 points). A high percentage of patients (54.7%) had stage 4 renal disease, and diabetes mellitus (DM) and nephroangiosclerosis were the main causes of kidney disease. The patient characteristics are shown in Table 1.

### 3.2. Mortality Outcome

Fifty patients died during the study period (8 years), 28 (56%) were men and 22 (44%) women. Mortality causes were cardiovascular disease (43.1%), tumors (17.6%), cerebral hemorrhage (11.8%), infections (11.8%), and other causes (15.7%). Different sociodemographic and clinical variables were analyzed in deceased patients and in those who survived and the results are shown in Table 2. A higher mortality was observed in patients aged 75–84 years and in those who had attended the CKD unit visits for less than 6 months. The values for body composition and biochemical parameters were better in those who survived.

Since there are no specific cut-off points for kidney disease in the different varieties of the STS test, cut-off points for the study sample were calculated by means of ROC curves, using the SPPB test as a reference. We obtained the following cut-off points: for STS5 the cut-off was 12.5 s (AUC: 0.885; 95% CI: 0.832–0.938; *p*-value < 0.001); for STS10 the cut-off was 27.5 s (AUC: 0.879; 95% CI: 0.823–0.934; *p*-value < 0.001); for STS30 the cut-off was 11 repetitions (AUC: 0.889; 95% CI: 0.837–0.941; *p*-value < 0.001); and for STS60 the cut-off was 19 rep (AUC: 0.839; 95% CI: 0.776–0.941; *p*-value < 0.001).

**Figure 1:**
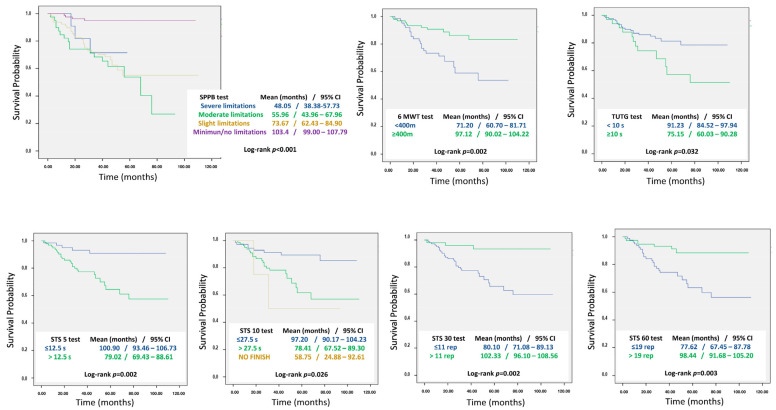
Survival results in relation with functionality according Kaplan–Meier curves. Kaplan–Meier curves of the mortality rates for the SPPB test, 6MWT, TUTG and four versions of STS tests Abbreviations: SPPB = Short Physical Performance Battery; 6MWT = 6 Minutes Walk Test; TUTG = Timed Up and Go; STS = Sit to Stand; CI = confidence interval; s = seconds; rep = repetitions.

Using Kaplan–Meier curves, survival was determined according to the functional capacity tests results, showing that those patients with better test scores had a longer survival time. The results are shown in Figure 1.

In Table 3, it can be seen that the patients who survived had a significantly better median value across the different functionality tests, as well as in terms of the percentage of patients who were within the normal range for each test.

Survival cut-off points for each of the tests were calculated using ROC curves, and the results are shown in Table 4.

In the multivariate COX regression analysis, we obtained the two best models associated with a higher survival. In both models, inflammation and lower functionality appeared as risk factor increasing mortality. In model 1 high albumin values and in model 2 a higher intracellular water percentage were associated with lower mortality (Table 5).

Using Cox multivariate proportional hazards analysis, after adjusting for potential confounders, in model 1, several parameters, such as SPPB (functional capacity), albumin (nutritional biochemical parameter), and CRP (inflammation parameter), and in model 2, factors such as SPPB, CRP and %IBW (like muscular parameter), were significantly associated with the risk of all-cause mortality in CKD patients.

## 4. Discussion

The aim of the study was to determine the influence of functional capacity on mortality in patients with ACKD. The results showed that low scores in the SPPB test, a shorter distance covered in the 6MWT, a longer execution time in the TUTG or in the STS5 and STS10 tests, and a lower number of squats performed in the STS30 and STS60 tests were associated with higher mortality. We also found that those who died were older on average, frail or pre-frail, and had more co-morbidities, as well as a poorer nutritional status and/or poorer body composition.

Survival predictors, including SPPB test results, nutrition-inflammation parameters (albumin and CRP), and %IBW, were identified in the multivariate COX regression analysis as body composition parameters that indicated improved muscle mass [30,31].

Functionality is contingent upon nutritional status [32]. A poor nutritional status exacerbates body composition by reducing muscle mass, which is significantly correlated with functional capacity, thereby promoting the development of protein-energy wasting [32,33]. Consequently, patients with a lower nutritional status, a poor body composition, and, as a result, a lower level of functionality experience a higher mortality rate [34]. This explains why patients with a lower nutritional status, a poorer body composition, and, as a result, a lower level of functionality experience a higher mortality rate [35]. The patients who passed away in our study exhibited reduced levels of albumin and intracellular body water by BIA in multivariate COX regression, in relation to malnutrition stage.

The group of deceased patients exhibited a decrease in muscle strength as measured by hand grasp strength. HGS is not a functionality test. However, the reduced muscle strength detected by this test indicates a likely increased risk of fragility and sarcopenia, which are also significant predictors of mortality in patients with chronic kidney disease [36,37,38].

Although the SPPB test is primarily designed for geriatric patients, it appears to be a more accurate predictor of mortality than the other tests that were analyzed. Ribeiro Silva et al. [39] have reviewed the utility of this test in a variety of studies, which include a wide range of pathologies. In patients with a score below 10 points, these studies demonstrated an increase in mortality. However, in order to ensure that this test is utilized accurately, it is necessary to establish specific cut-off points for each pathology. We have established a 7.5-point cut-off for ACKD patients in our study. In a 2016 meta-analysis conducted by Pavasini et al. [40], which encompassed 17 investigations with over 50 ACKD patients, the cut-off point was established at 7–9 points (mild limitations). Not only did they observe an increase in mortality in geriatric patients, but also in men, younger patients, and those who were diabetic. In our study, although mortality was higher in elderly patients, the young patients who died also had SPPB < 10, as reported by other authors [41,42].

Other studies, such as that conducted by Lattanzio et al. in 2015 [43], have reported comparable outcomes. In their study, they assessed mortality at one year in a cohort of 487 geriatric patients. In contrast to the four categories previously described, the test results in this study were categorized into three groups: 0–4 points, 5–8 points, and 9–12 points. The mortality rate was 74.2% for patients with ≤4 points, 19.7% for those with 5–8 points, and 6.1% for those with ≥9 points. The mortality percentages would be significantly different in our study if this classification were implemented: 18% for points ≤4, 54% for 5–8 points, and 28% for points ≥9. This is likely attributable to the fact that patients who had attended CKD unit visits for more than six months were encouraged to engage in physical activity and were provided with dietary advice. This could also account for the fact that the maximum mortality rate occurs within the first six months of the ACKD consultation, as the patient would not have received these recommendations yet.

A reduction in walking speed is highly correlated with frailty, which significantly elevates the likelihood of mortality. A reduction in walking velocity below 0.8 m per second [44] and covering a distance of less than 400 m during the 6MWT are linked to increased death rates [45,46]. The threshold value that provided the highest level of accuracy in predicting death in our investigation was 367.5 m. The findings are consistent with previous research, such as Roshanravan et al., 2013 [47], who identified a threshold of <350 m, or Ciudad A et al., 2018 [48], in which the mortality rates were higher among patients who traveled a distance <355 m.

An individual is classified as sedentary in the general population if they walks less than 5000 steps per day [49]. Engaging in physical activity at a level of 5000 to 7500 steps is classified as low activity. However, a study conducted by Paluch et al. in 2021 set the maximum limit of this group at 7000 steps per day [50]. An individual is deemed active if they exceeds 7500 steps per day. Patients experience the optimal benefits by achieving a minimum of 10,000 steps per day, with some studies suggesting an even higher threshold of 12,500 steps per day [51]. For every additional 1000 steps taken per day, there is a reduction in mortality caused by cardiovascular issues and diabetes, which are common comorbidities in CKD. Thus, incorporating modest physical exercise into a patient’s daily routine can be advantageous in mitigating their mortality risk [52,53].

This was demonstrated in the 2019 study conducted by Clarke et al. [54], which assessed the survival rates of patients with chronic kidney disease (without undergoing dialysis). It was shown that patients who walked regularly, particularly those who had a step speed of ≥1.34 m/s, had a 63% lower chance of mortality. Previous research has examined the advancement of kidney disease in patients who engaged in physical activity following a heart attack. These investigations found that the course of the disease was slower in patients who walked more steps per day [55]. Although we did not measure the exact number of steps taken each day in our study, we found that patients who performed better on the 6MWT test and hence had a greater daily step count, had a reduced risk of mortality [56].

The 2020 retrospective study conducted by Joo YS et al. [57] examined the likelihood of acquiring chronic kidney disease (CKD) by assessing the TUG test score in a sample of 30,871 adult patients aged 66 years or older. The study utilized data from the National Health Insurance Service National Sample Cohort Database (NHIS-NSC DB) of Korea. The data revealed a correlation between the duration of the TUTG and an escalation in the incidence of CKD and mortality rate. The researchers separated the population into three tertiles. They found that tertile 3, which had a cut-off point set at 14.9 ± 6.3 s, had a higher risk of mortality. When determining the optimal duration for optimum sensitivity and specificity using ROC curves, we found a cut-off point of 7.5 s, which is lower than the cut-off point reported by Joo YS et al. This point could be inserted in the 1 or 2 tercile of the described study.

The STS test in renal disease does not have any documented cut-off points. Like other tests, specific cut-off points would need to be specified for each pathology [58]. Our study involved calculating cut-off points for each version of the STS and determining the greatest sensitivity and specificity for survival using the SPPB test as a reference. We found that the scores obtained were remarkably similar across all versions of the STS. The 2022 study conducted by Höglund J. et al. [59] examined the mortality rates of patients diagnosed with Chronic Obstructive Pulmonary Disease (COPD) by utilizing the 6MWT and STS30 tests. A cut-off point of 11 squads was determined, although our study had a value of 10. Regardless of the scenario, the 6MWT test demonstrated superior efficacy in predicting the risk of mortality. In a separate study including patients with COPD [60], a cut-off value of 15.98 s was established for the STS5 test. In our study, however, the cut-off point was chosen at 13.5 s.

Obtaining a favorable result in any of the functionality tests indicates possessing a high level of functional capacity. Multiple studies have demonstrated the correlation between effective functionality and decreased death rates [61]. As this study is based on typical clinical practice, additional investigations are required to establish the specific type, frequency, and intensity of the condition, as well as to develop a standardized procedure for assessing chronic kidney disease (CKD) (as there is currently no agreement on the CKD assessment protocol).

## 5. Conclusions

This study shows how functional capacity influences mortality. The patients with ACKD who exhibit a decline in functional capacity are at an elevated risk of mortality. The SPPB test is suitable for assessing functional capacity across all age groups of CKD patients, better than other tests. Furthermore, our study suggests that this test is the most reliable predictor of the risk of mortality when compared to the other tests that were examined.

## Figures and Tables

**Table 1:** General characteristics of the study population.

	Overall *n* = 225	Men *n* = 148 (65.8%)	Women *n* = 77 (34.2%)	*p*-Value
Age (yrs, mean ± SD)	70.65 ± 11.97	69.85 ± 11.16	72.19 ± 13.33	0.189
BMI	27.00 ± 4.91	27.62 ± 4.38	27.23 ± 5.82	0.574
Age in groups (yrs, mean ± SD)				
<55	48.29 ± 5.57	48.00 ± 5.27	48.72 ± 6.21	<0.001
55–64	59.50 ± 2.73	59.85 ± 2.78	58.55 ± 2.45	
65–74	70.21 ± 2.82	70.39 ± 2.53	69.66 ± 3.61	
75–84	79.19 ± 3.11	78.8 ± 3.03	79.75 ± 3.21	
≥85	87.65 ± 2.66	87.85 ± 3.33	87.53 ± 2.36	
Age group *n* (%)				
<55	27 (12)	16 (10.8)	11 (14.3)	0.012
55–64	36 (16)	27 (18.2)	9 (11.7)	
65–74	61 (27.1)	46 (31.1)	15 (19.5)	
75–84	81 (36)	52 (35.1)	29 (37.7)	
≥85	20 (8.9)	7 (4.7)	13 (16.9)	
ACKD stage *n* (%)				
Stage 3B	19 (8.4)	9 (6.1)	10 (13)	0.102
Stage 4	123 (54.7)	87 (58.8)	36 (46.8)	
Stage 5 (ND)	83 (36.9)	52 (35.1)	31 (40.3)	
ACKD vintage *n* (%)				
<6 months	147 (65.3)	97 (65.5)	50 (64.9)	0.750
6–12 months	29 (12.9)	21 (14.2)	8 (10.4)	
>12 months	49 (21.8)	30 (20.3)	19 (24.7)	
Comorbidity (mean ± SD/Median)				
Charlson Index	6.46 ± 1.92/6	6.56 ± 1.88/7	6.27 ± 1.99/6	0.298
DM *n* (%)				
Yes	98 (43.6)	73 (49.3)	25 (32.5)	0.016
No	127 (56.4)	75 (50.7)	52 (67.5)	

Abbreviations: yrs = years; SD = standard deviation; BMI = body mass index; ACKD = advanced chronic kidney disease; ND = no dialysis; DM = diabetes mellitus.

**Table 2:** Comparison of sociodemographic and clinical parameters between survivors and non-survivors.

		Exitus *n* = 50	No Exitus *n* = 175	*p*-Value
Sex *n* (%)	Male	28 (56)	120 (68.6)	0.098
	Female	22 (44)	55 (31.4)	
Age (mean ± SD)		79.02 ± 7.39	68.26 ± 11.97	<0.005
Age range *n* (%)				
<55		0 (0)	27 (15.4)	<0.001
55–64		2 (4)	34 (19.4)	
65–74		10 (20)	51 (29.1)	
75–84		28 (56)	53 (30.3)	
≥85		10 (20)	10 (5.7)	
Time in CKD unit *n* (%)				
<6 months		35 (70)	112 (64)	0.293
6–12 months		8 (16)	21 (12)	
>12 months		7 (14)	42 (24)	
Charlson index (mean ± SD)		7.64 ± 1.61	6.12 ± 1.88	<0.001
Fried criteria (mean ± SD)		2 ± 1.42	0.87 ± 1.11	<0.001
No frail *n* (%)		7 (14)	85 (48.6)	<0.001
Pre frail *n* (%)		26 (52)	67 (38.3)	
Frail *n* (%)		34 (17)	23 (13.1)	
Body Composition (mean ± SD)				
Phase angle		3.81 ± 0.96	4.35 ± 1.10	0.002
Na/K		1.52 ± 0.49	1.34 ± 0.41	0.014
%BCM		39.16 ± 8.06	43.05 ± 8.03	0.003
%TBW		53.90 ± 8.68	53.15 ± 7.00	0.529
%IBW		40.57 ± 7.56	44.26 ± 7.57	0.003
%EBW		59.42 ± 7.56	55.73 ± 7.57	0.003
%FM		31.44 ± 10.51	31.16 ± 8.58	0.845
%FFM		68.56 ± 10.51	68.83 ± 8.59	0.846
%MM		32.06 ± 9.20	32.99 ± 7.32	0.460
ASMM		17.4 ± 14	19.55 ± 4.74	0.001
BCMI		7.18 ± 2.00	8.11 ± 1.97	0.004
BMI		27.11 ± 5.72	27.60 ± 4.67	0.541
Laboratory parameters (mean ± SD)				
Albumin (g/dL)		4.05 ± 0.42	4.26 ± 0.40	0.002
Prealbumin (mg/dL)		24.96 ± 6.12	28.71 ± 7.97	0.004
CRP (mg/dL)		1.08 ± 1.79	0.58 ± 1.25	0.028
Lymphocytes (miles/mm^3^)		1828.60 ± 911.83	2114.16 ± 929.73	0.056
Transferrin (mg/dL)		216.50 ± 49.47	220.84 ± 52.27	0.602
HB (g/dL)		11.94 ± 1.39	12.33 ± 1.57	0.120
CKD-EPI eGFR (mL/min/1.73 m^2^)		16.39 ± 5.78	19.47 ± 7.99	0.011

Abbreviations: SD = standard deviation; CKD = chronic kidney disease; BCM = body cell mass; TBW = total body water; IBW = intracellular body water; EBW = extracellular body water; FM = fat mass; FFM = free fat mass; MM = muscle mass; BCMI = body cell mass index; BMI = body mass index; ASMM = appendicular skeletal muscle mass; CRP = C-reactive protein; HB = hemoglobin, CKD-EPI = chronic kidney disease epidemiology collaboration equation; eGFR= estimate glomerular filtration rate.

**Table 3:** Differences in functional capacity between survivors and patients who passed away.

	Exitus *n* = 50	No Exitus *n* = 175	*p*-Value
SPPB			
SPPB (mean ± SD)	6.80 ± 2.24	8.99 ± 2.77	<0.001
Severe limitations *n* (%)	3 (23.1)	10 (76.9)	<0.001
Moderate limitations *n* (%)	17 (42.5)	23 (57.5)	
Slight limitations *n* (%)	26 (32.5)	54 (67.5)	
Minimum/no limitations *n* (%)	4 (4.3)	88 (95.7)	
6MWT			
6MWT (mean ± SD)	369.53 ± 50.42	422.87 ± 95.73	<0.001
<400 m *n* (%)	21 (32.8)	11 (11.5)	<0.001
>400 m *n* (%)	43 (67.2)	85 (88.5)	
TUTG			
TUTG (mean ± SD)	9.258 ± 3.08	8.08 ± 2.05	0.013
<10 s *n* (%)	20 (62.5)	107 (863.6)	0.008
>10 s *n* (%)	12 (37.5)	21 (16.4)	
STS5			
STS5 (mean ± SD)	17.78 ± 6.59	14.18 ± 4.94	0.006
≤12.5 s *n* (%)	5 (7.7)	60 (92.3)	<0.001
>12.5 s *n* (%)	27 (28.4)	68 (71.6)	
STS10			
STS10 (mean ± SD)	34.03 ± 10.42	29.29 ± 8.35	0.026
≤27.5 s *n* (%)	8 (10.5)	68 (89.5)	0.006
>27.5 s *n* (%)	22 (27.5)	58 (72.5)	
STS30			
STS30 (mean ± SD)	8.78 ± 2.39	10.78 ± 3.04	<0.001
≤11 rep *n* (%)	29 (27.1)	78 (72.9)	0.001
>11 rep *n* (%)	3 (5.7)	50 (94.3)	
STS60			
STS60 (mean ± SD)	15.87 ± 5.21	20.53 ± 6.35	<0.001
≤19 rep *n* (%)	25 (30.9)	56 (69.1)	0.001
>19 rep *n* (%)	7 (8.9)	72 (91.1)	
Handgrip Strength			
HGS Right (mean ± SD)	20.56 ± 7.72	28.12 ± 10.71	<0.001
HGS Left (mean ± SD)	19.24 ± 8.10	25.37 ± 10.54	<0.001

Abbreviations: SD = standard deviation; SPPB = Short Physical Performance Battery; 6MWT = 6-minutes walking march test; TUTG = timed up and go; STS = Sit to Stand; HGS = handgrip strength.

**Table 4:** Cut-off points of functional capacity for mortality.

	Cut-Off	Sensitivity/Specificity	AUC	95% CI	*p*-Value
SPPB (pnts)	7.5	74%/66%	0.745	0.678–0.812	<0.001
6MWT (m)	367.5	74%/50%	0.696	0.611–0.781	0.001
TUTG (s)	7.7	65%/51%	0.639	0.524–0.754	0.015
STS5 (s)	13.5	81%/56%	0.721	0.630–0.812	<0.001
STS10 (s)	28.5	76%/57%	0.664	0.558–0.769	<0.001
STS30 (rep)	10	65%/62%	0.699	0.605–0.792	0.001
STS60 (rep)	19	61.7%/71.9%	0.722	0.628–0.817	<0.001
HGS Right (kg)	26	56%/78%	0.704	0.630–0.777	<0.001
HGS Left (kg)	22	59%/66%	0.672	0.593–0.751	<0.001

Abbreviations: AUC = area under de curve; CI = confidence intervals; m = meters; pnts = points; rep = repetitions; s = second.

**Table 5:** Multivariate COX regression of factors affecting functionality.

	Model 1		Model 2	
	HR (95% CI)	*p*-Value	HR (95% CI)	*p*-Value
SPPB (points)	0.764 (0.683–0.855)	<0.001	0.778 (0.695–0.872)	<0.001
Albumin (g/dL)	0.456 (0.210–0.992)	0.048	-	-
CRP (mg/dL)	1.246 (1.014–1.531)	0.036	1.333 (1.104–1.610)	0.003
%IBW	-	-	0.935 (0.900–0.971)	0.001

Abbreviations: HR = hazard ratio; CI = confidence interval; SPPB = Short Physical Performance Battery; CRP = C-reactive protein; IBW = intracellular body water.

## Data Availability

Data is contained within the article.
